# Digitale Informationsmaterialen über Demenz – Eine explorative inhaltsanalytische Betrachtung

**DOI:** 10.1007/s00103-024-03893-7

**Published:** 2024-05-28

**Authors:** Dominik Daube, Doreen Reifegerste

**Affiliations:** 1 Friedrich-Schiller-Universität Jena, Jena, Deutschland; 2 Professur für Gesundheitskommunikation, Institute for Planetary Health Behaviour (IPB), Universität Erfurt, Nordhäuser Str. 63, 99089 Erfurt, Deutschland; 3 Bernhard-Nocht-Institut für Tropenmedizin, Hamburg, Deutschland; 4 Fakultät für Gesundheitswissenschaften, Universität Bielefeld, Bielefeld, Deutschland

**Keywords:** Demenzen, Risikofaktoren, Prävention, Onlinerecherche, Entscheidungsfindung, Dementia, Risk factors, Prevention, Online research, Decision-Making

## Abstract

**Hintergrund:**

Eine Demenzdiagnose bedeutet eine hohe Belastung für die Betroffenen und ihre Angehörigen. Häufig müssen schwierige Entscheidungen getroffen werden. Idealerweise treffen Menschen mit einer neuen Demenzdiagnose informierte Entscheidungen gemeinsam mit Angehörigen. Im Vorfeld einer informierten Entscheidung sind digitale Informationsmaterialien eine wichtige niederschwellige Quelle. Sie sollten möglichst umfassend über Demenz aufklären, sowohl zur Primärprävention (Risikofaktoren für Demenzentstehung) als auch zu späteren Behandlungsmöglichkeiten. Auch über vorsorgliche Maßnahmen, die spätere Entscheidungen erleichtern können (bspw. Patient*innenverfügung, Vorsorgevollmacht), sollte informiert werden. Einen umfassenden Überblick über die diversen Onlineangebote zu Demenzerkrankungen gibt es bisher nicht.

**Methode:**

Daher untersucht diese Studie im deutschen Sprachraum mittels einer innovativen systematischen Suchstrategie, welche demenzbezogenen Informationsangebote es digital für welche Zielgruppen gibt und wie diese Materialien inhaltlich hinsichtlich Risikofaktoren und Behandlungsmaßnahmen sowie Entscheidungsunterstützungen aufbereitet sind. Dieser methodische Ansatz ist neu und wird daher ausführlich vorgestellt und diskutiert.

**Ergebnisse:**

Die Ergebnisse zeigen, dass die meisten Materialien für Menschen mit Demenz sowie deren Angehörige aufbereitet sind. Häufig werden Behandlungsmaßnahmen thematisiert, ohne die Risikofaktoren zu erklären, auf die diese Maßnahmen abzielen. Auf präventive Maßnahmen zur Entscheidungsunterstützung wird kaum eingegangen.

**Diskussion:**

Die unausgewogene Darstellung kann dazu führen, dass einzelne Maßnahmen (für Laien) schwer nachvollziehbar sind oder Risikofaktoren falsch eingeordnet werden. Wichtige Implikationen werden abgeleitet.

## Abstract (en)

**Background:**

A diagnosis of dementia places a heavy burden on those affected and their families. Often, difficult decisions must be made. Ideally, people with a new dementia diagnosis make informed decisions together with family members. Digital informational materials can be an important low-threshold resource for making informed decisions. They should provide comprehensive information about dementia, including both primary prevention (risk factors for the onset of dementia) and later treatment options. They should also cover precautionary measures that can make future decisions easier (e.g., advance directives, power of attorney). However, there is currently no comprehensive overview of the various online resources for dementia-related information.

**Methods:**

This study explores digital informational materials on dementia for different target groups in the German-speaking area using an innovative systematic search strategy. It examines how these materials are structured in terms of risk factors, treatment options, and decision support. This methodological approach is new, so it is described and discussed in detail.

**Results:**

The results show that most materials are intended for people with dementia and their families. Treatment measures are often discussed without explaining the risk factors they are meant to address. There is little focus on preventive measures that could support decision-making.

**Discussion:**

The unbalanced presentation could lead to certain measures being difficult to understand (for laypeople) or to misinterpretation of risk factors. Important implications are drawn from these findings.

## Einleitung

Die Zahl der Menschen mit Demenz weltweit wird sich bis 2050 schätzungsweise verdreifachen. Auch in Deutschland wird ein Anstieg von aktuell 1,8 Mio. auf rund 2,8 Mio. Menschen mit Demenz bis 2050 prognostiziert [1–4]; aktuelle Studien kalkulieren einen etwas niedrigeren Anstieg [3]. Dementsprechend spielt auch die Prävention eine wichtige Rolle, sowohl um primärpräventiv Risikofaktoren zu reduzieren bzw. diagnostizierte Erkrankungen zu behandeln (gesundheitsrelevante Maßnahmen) als auch um darüber aufzuklären, welche Maßnahmen anstehende Entscheidungen nach einer Diagnose unterstützen und erleichtern können (entscheidungsbezogene Faktoren). Allerdings ist in der Bevölkerung noch vielfach unbekannt, dass und mit welchen Methoden Demenzen vorgebeugt werden kann [5]. Folglich ist es notwendig, Informationen über Demenzen bereitzustellen und im Rahmen koordinierter Public-Health-Maßnahmen (öffentlicher Gesundheitskampagnen) zu verbreiten. Gleichzeitig sind individualisierte Interventionsmaßnahmen notwendig, wozu beispielsweise speziell für die jeweilige Zielgruppe aufbereitete (Targeting, Tailoring) Informationsmaterialien zählen [4]. Diese Informationen sind generell für Menschen in jedem Alter relevant, da die Risikofaktoren sich über die gesamte Lebensspanne verteilen [4]. Zudem betonen Livingston et al., dass es nicht nur für (potenziell) Betroffene wichtig ist, Informationen über die Entstehung und den Verlauf von Demenzen bereitzustellen und über die Eigenschaften von Demenzen aufzuklären, sondern auch ihre Angehörigen zu adressieren und „care for family carers“ [4] sicherzustellen. Somit sollten mit den Präventionsinformationen breite Bevölkerungsschichten erreicht werden.

Informationsmaterialien sind eine wichtige Möglichkeit, um laienverständliche, qualitativ hochwertige Informationen über Gesundheitsthemen für breite Bevölkerungsschichten zur Verfügung zu stellen [6–8]. Diese können sowohl als Printprodukte (bspw. Broschüren oder Informationsblätter) als auch digital (Informationswebsites, Online-Enzyklopädien) sowie hybrid (digital abrufbare Broschüren/Informationsblätter) vorliegen. Dabei sind gerade digitale und hybride Formate niederschwellig erreichbar sowie zeit- und ortsunabhängig verfügbar und werden häufig genutzt [9, 10]. Sie sind auch für ältere Zielgruppen (und ihre Angehörigen) eine relevante Informationsquelle [11]. Daher werden in dieser Studie digitale und hybride Formate fokussiert.

Bisherige Inhaltsanalysen haben Materialien sehr spezifisch mit sehr engen Inklusionskriterien (z. B. nur konkretes Krankheitsbild, konkrete Anbieter*innen) untersucht, z. B. [12–14]. Eine systematische Untersuchung, welche Informationsmaterialien digital oder hybrid (im deutschsprachigen Raum) verfügbar sind und welche Inhalte dort vermittelt werden, gibt es bislang nicht. Daher adressiert die vorliegende Studie diese Forschungslücke im Kontext von Demenzen und untersucht systematisch (a) formal, für welche Zielgruppen Materialien online verfügbar sind, und (b) inhaltlich, ob Risikofaktoren und Behandlungsoptionen sowie entscheidungsbezogene Faktoren repräsentiert werden. Inhaltlich werden in dieser Studie gesundheitsrelevante und entscheidungsbezogene Faktoren unterschieden, die nachfolgend eingeordnet werden.

### Gesundheitsrelevante Faktoren.

Dahlgren und Whitehead [15, S. 11] definieren 3 Ebenen, über welche sie zentrale Determinanten von Gesundheit strukturieren und gesundheitsrelevante Maßnahmen ableiten:

- Individuum (Alter, Geschlecht, Lifestyle),
- soziales Netzwerk (Unterstützung),
- Gesellschaft (Sozioökonomie, Kultur, Umwelt).

Als „gesundheitsrelevant“ werden dabei Faktoren bezeichnet, die sich auf den individuellen Gesundheitszustand auswirken (Begriffsabgrenzung z. B. [16]). Traditionell werden innerhalb der gesundheitsrelevanten Faktoren Risikofaktoren von Behandlungsoptionen unterschieden (z. B. [17, 18]). Für eine effektive (Krankheits‑)Prävention ist es essenziell, sowohl über Risikofaktoren als auch über Behandlungsoptionen zu informieren – also, hier am Beispiel Demenz, einerseits Risikofaktoren zu kommunizieren, welche die Wahrscheinlichkeit erhöhen, ein Demenzsyndrom auszubilden, aber andererseits auch Möglichkeiten aufzuzeigen, wie man diesen Risikofaktoren vorbeugen kann bzw. eine Demenz behandeln kann.

In der Kommunikation kann bewusst oder unbewusst eine Fokussierung auf einzelne Themen stattfinden, die als „Framing“ (Rahmung) von Inhalten bezeichnet wird [19]. Dadurch wird eine bestimmte Deutung der Information impliziert. Ein konkreter Anwendungsbereich ist das „Responsibility Framing“, hier geht es um die kommunizierten Verantwortlichkeiten, – d. h., beispielsweise bezogen auf die vorliegende Arbeit, auf welchen Ebenen Verantwortung für Risikofaktoren und Behandlungsoptionen inhaltlich zugeschrieben wird [20, 21]. Die einzelnen gesundheitsrelevanten Faktoren [4, 22] lassen sich im Wesentlichen auf die 3 genannten Ebenen aggregieren: auf die individuelle Ebene (d. h. die einzelnen (potenziell) Betroffenen), die Ebene des sozialen Netzwerks (d. h. Angehörige/Freund*innen) und die gesellschaftliche Ebene [23, 24].

### Entscheidungsbezogene Faktoren.

Diese haben keinen direkten Einfluss auf den Gesundheitszustand, sondern können Entscheidungen unterstützen, die die Gesundheit fördern. Bezogen auf Demenz müssen entweder die Menschen mit Demenz selbst Entscheidungen treffen, sofern sie juristisch dazu in der Lage sind, oder Angehörige entscheiden stellvertretend. Unterscheiden lassen sich innerhalb der entscheidungsbezogenen Faktoren formelle (juristisch bindende) und informelle Maßnahmen. Letztere sind individuell zu ergreifen und nicht an Vorgaben der Legislative gebunden.

Zu *informellen* Faktoren zählen bspw. Maßnahmen wie klärende (präventive) Gespräche mit Angehörigen und Freund*innen über gesundheitsbezogene Maßnahmen und den eigenen Behandlungswillen. Die *formellen* Maßnahmen erfordern hingegen festgelegte formale Schritte und gehen mit einer juristischen Bindung einher. So gibt es bspw. Vorgaben, wie gewünschte Maßnahmen in einer Patient*innenverfügung beschrieben sein müssen (z. B. widerspruchsfreie Formulierungen, konkrete Situationsbeschreibung), um juristisch bindend und anwendbar zu sein [25]. Dazu sind Informationen essenziell, da diese formellen Maßnahmen proaktiv von den (potenziell) betroffenen Menschen initiiert und umgesetzt werden müssen, d. h., sie müssen die Möglichkeiten kennen und auch wissen, welche Schritte zu unternehmen sind.^1^

In Anlehnung an die Struktur der aufbereiteten Informationsmaterialien und der darin angesprochenen Zielgruppen sowie der beschriebenen kommunizierbaren Inhalte stellen wir folgende Forschungsfragen:

### FF1:

Welche Zielgruppen werden in deutschsprachigen Informationsmaterialien über Demenzen adressiert und auf welche zusätzlichen Informationsangebote wird für die Zielgruppen verwiesen?

### FF2:

Welche a) gesundheitsrelevanten und b) entscheidungsbezogenen Faktoren werden in demenzbezogenen Informationsmaterialien kommuniziert?

### FF2a:

Auf welchen Ebenen (individuell, soziales Netzwerk, gesellschaftlich) werden gesundheitsrelevante Risikofaktoren und Behandlungsoptionen erwähnt?

### FF2b:

Welche informellen und formellen entscheidungsbezogenen Maßnahmen werden genannt?

## Methoden

Um die Forschungsfragen zu beantworten, wurde eine quantitative Inhaltsanalyse von deutschsprachigen, frei zugänglichen Informationsmaterialien über Demenzen durchgeführt.

### Stichprobenziehung

Eine besondere Herausforderung stellte die Stichprobenziehung dar, um digitales Informationsmaterial möglichst breit und repräsentativ erfassen zu können. Wie einleitend beschrieben, haben bisherige Studien Materialien sehr spezifisch mit engen Inklusionskriterien untersucht, was die Datengewinnung erleichterte. Somit konnten diese Verfahren nicht für die vorliegende Studie adaptiert werden. Daher wurde ein innovatives systematisches Vorgehen in Anlehnung an die Standards von Systematic Reviews [26] entwickelt, welches nachfolgend ausführlich vorgestellt wird. Ziel der Strategie war es, eine Online-Informationssuche von Laien zu simulieren und Materialien möglichst breit und repräsentativ zu beschaffen, die sowohl von staatlichen Institutionen und wissenschaftlichen Einrichtungen als auch von Gesundheits- und Pharmakonzernen sowie (privaten) Website-Anbieter*innen stammen können. Auch die potenzielle Verzerrung der Rechercheergebnisse durch unterschiedliche (intransparente und unbekannte) Suchalgorithmen (inkl. Cookie-Settings) stellte eine Schwierigkeit dar. Wie dieser begegnet wurde, wird nachfolgend dargestellt – gleichzeitig ist dies auch eine Limitation, die auch in der Realität bei der Informationssuche auftritt.

**Abb. 1:**
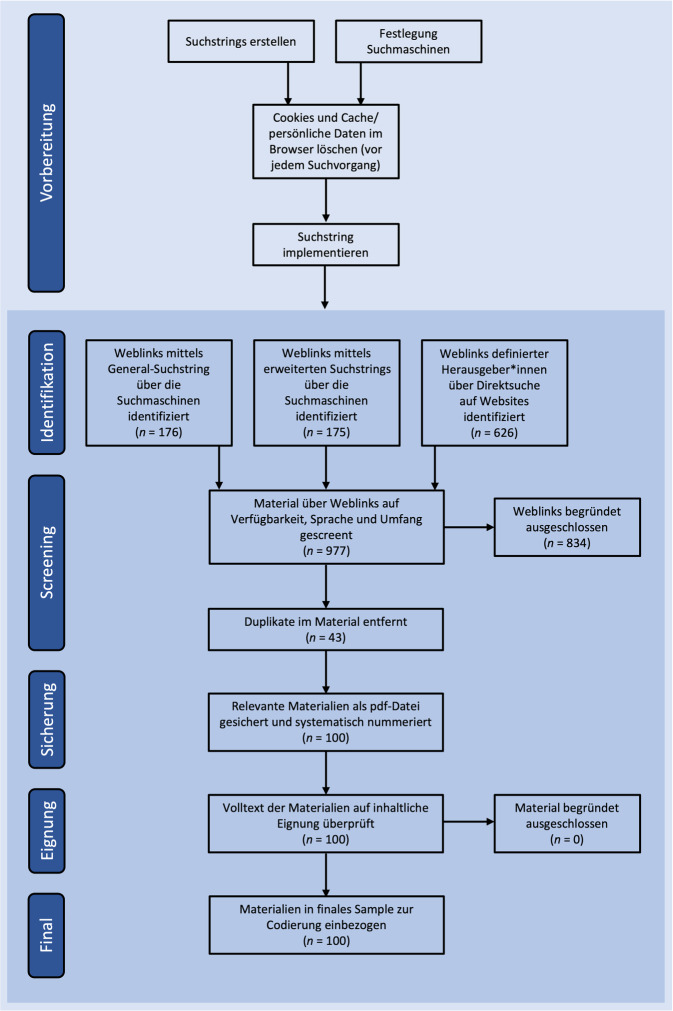
Systematische Suchstrategie für die Stichprobenziehung, adaptiert und erweitert nach dem „PRISMA Flow Diagram“ für Systematic Reviews [26]. (Quelle: eigene Abbildung)

Insgesamt 3 Personen recherchierten die Materialien im Juni und Juli 2021 und glichen die Ergebnisse ab.^2^ Abb. 1 zeigt das Vorgehen schematisch.

Zunächst wurde ein *General-Suchstring* erstellt:

′((Info* OR Aufklärung* OR Angebot* OR Broschüre OR Material OR Flyer OR Kampagne*) AND (Alzheimer* OR Demenz*))′

Im ersten Schritt wurden in den jeweiligen Browsern Cookies und persönliche Daten (Cache, Verlauf) gelöscht. Der Suchstring wurde anschließend in insgesamt 5 Internet-Suchmaschinen zur Recherche (Algorithmen, Rankings) implementiert (Google, Bing, Qwant, MetaGer, Fireball). Es wurden jeweils die ersten 50 Suchergebnisse gesichert und anhand folgender Inklusionskriterien gescreent:

- Sprache: Deutsch,
- Verfügbarkeit: kostenlos und online frei abrufbar,
- Umfang: maximal 20 Seiten (bei Websites im A4-Format als PDF-Datei gesichert); Begründung: Nur Materialien inkludieren, die kompakt aufbereitet wurden und als Informationsmaterial geeignet erscheinen, längere und umfangreichere Überblickswerke dienen nicht einer ersten Information, sondern setzen deutlich vertiefte Auseinandersetzung mit der Thematik voraus.

Dopplungen wurden entfernt und einfach erfasst. Alle relevanten Ergebnisse wurden anschließend als PDF-Datei gesichert.

Parallel zum *General-Suchstring* wurde ein zweiter *erweiterter* Suchstring implementiert, um gezielt Ergebnisse aus der Medizin- und Pharmaindustrie zu erfassen:

′AND (Pharma* OR Medikament)′

Hier wurde wie mit dem *General-Suchstring* verfahren. Um zusätzlich zu den randomisiert erfassten Ergebnissen auch die Materialien zu inkludieren, die unter Umständen durch die Suchalgorithmen der Suchmaschinen nicht berücksichtigt werden, aber auf die bei der realen Informationssuche durch Empfehlungen von Expert*innen verwiesen wird, wurde eine intensivierte Suche ergänzt. Es wurden Datenbanken und Websites systematisch und nach transparenten harten Kriterien durchsucht:

- Relevanz Herausgeber*in: Ranking im bundesdeutschen Vergleich, z. B. Top 5 auf dem Tätigkeitsgebiet, gemessen an a) wirtschaftlicher Bedeutung (Umsatz oder Mitgliederzahl) oder b) Alexa-Traffic Rank (weltweite Website-Popularität nach Besucher*innenzahlen),
- Sprache: Deutsch,
- Umfang: maximal 20 Seiten,
- Verfügbarkeit: kostenlos und online frei abrufbar.

Tab. 1 zeigt die Kategorien der Herausgeber*innen, inkl. der Kriterien.

**Tab. 1:** Herausgeber*innen-Kategorien und Kriterien für die Stichprobenziehung

Kategorie	Kriterien
Gesundheitswebsites	Top 30 nach Alexa-Traffic Rank
Internet-Enzyklopädien^a^	Top 10 nach Alexa-Traffic Rank
Kliniken	Top 10 nach Betten
Klinikunternehmen	Top 3 nach Umsatz in Deutschland
Krankenkassen	Top 10 nach Mitgliederanzahl
Pharmaunternehmen	Top 10 nach Umsatz in Deutschland

^a^Ohne primären Gesundheitsbezug, z. B. Wikipedia

Insgesamt wurden 977 recherchierte Links gescreent und hinsichtlich der Inklusionskriterien geprüft. 143 Materialien wurden heruntergeladen und um doppelte Materialien (*n* = 43) bereinigt, womit das finale Sample *N* = 100 beträgt.

### Messinstrument, Codierung und Datenanalysen

Die Codierung im Rahmen der Inhaltsanalyse fand auf Materialebene statt, die Kategorien wurden deduktiv aus der Theorie abgeleitet. Neben den formalen Kategorien (z. B. Publikationsform, Herausgeber*in, Umfang) wurden themenunabhängige Merkmale (Zielgruppe, Beratungsangebote) sowie themenabhängige Merkmale (z. B. Demenzart) codiert.

**Abb. 2:**
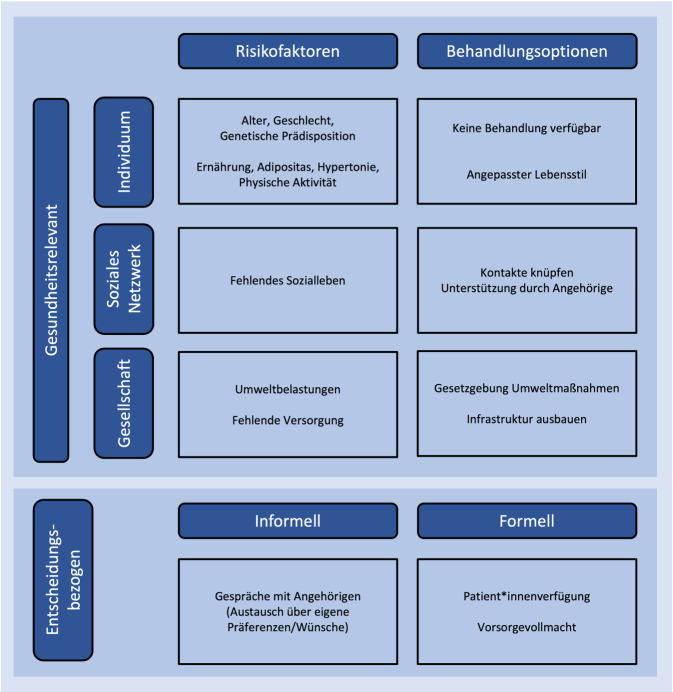
Inhaltsanalytische Kategorien zu gesundheitsrelevanten und entscheidungsbezogenen Faktoren mit beispielhaften Ausprägungen. (Quelle: eigene Abbildung)

Auf inhaltlicher Ebene wurden Risikofaktoren und Behandlungsoptionen auf den 3 Ebenen Individuum, soziales Netzwerk, Gesellschaft (siehe Einleitung) verschlüsselt und für diese Studie nur aggregiert ausgewertet. Zudem wurden die entscheidungsbezogenen formellen und informellen Maßnahmen codiert. Die inhaltsanalytischen Kategorien sind in Abb. 2 dargestellt. Bei der Codierung der gesundheitsrelevanten Faktoren wurde jeweils der Kontext berücksichtigt, bspw. ob die (potenziell) von Demenz Betroffenen selbst für die Pflege sozialer Kontakte (Unterkategorie „angepasster Lebensstil“) verantwortlich gemacht werden oder ob das soziale Netzwerk proaktiv auf das Sozialleben achten soll.

Vor der Codierung wurde eine Schulung mit den Codierenden und ein Pretest des Codebuchs durchgeführt, offene Fragen wurden besprochen. Anschließend wurde eine Probecodierung für den Reliabilitätstest mit einem Subsample durchgeführt. Die Intercoder-Reliabilität [27] wurde für alle relevanten Kategorien berechnet und ergab gute und interpretationsfähige Werte (alle *r*_*α*_ > 0,8). Die Beiträge wurden in der finalen Codierung von den 3 geschulten Codierenden codiert.

Die Datenanalysen wurden mit R/RStudio durchgeführt. FF1 wurde ausschließlich deskriptiv untersucht. FF2a und b wurden ergänzend inferenzstatistisch analysiert. Es wurden χ^2^-Tests durchgeführt, bei erwarteten Zellhäufigkeiten unter 5 wurde der exakte Test nach Fisher interpretiert.

Alle Auswertungen fanden aufgrund des geringen Forschungsstandes explorativ statt.

## Ergebnisse

### Informationsmaterialien

Insgesamt waren 73 Materialien als Websites rein digital aufbereitet, *n* = 27 waren auch als Printversion (Broschüre, Flyer, Poster) verfügbar. Mit Abstand am häufigsten traten privatwirtschaftliche Online-Gesundheitsportale (*n* = 34) als Herausgeber auf, gefolgt von eingetragenen Vereinen/gemeinnützigen Organisationen (*n* = 17). Staatliche Gesundheitsbehörden (*n* = 13), Kliniken (*n* = 12), Berufsverbände (*n* = 9) und Krankenkassen (*n* = 8) waren ebenfalls vertreten, nur selten kamen die Angebote dagegen von Pharmaunternehmen (*n* = 5), für *n* = 2 waren Herausgeber*innen nicht eindeutig zuzuordnen. Das Material umfasste durchschnittlich *M* = 1326,81 (*Md* = 903,50; *SD* = 1063,76) Wörter und wurde größtenteils in den letzten Jahren aktualisiert (50 % stammen aus 2018–2021; 17 % aus 2014–2017), in 33 % war kein eindeutiges Jahr der letzten Überarbeitung erkennbar. In über der Hälfte der Materialien (*n* = 63) werden Demenzen allgemein behandelt, 30 Materialien informieren spezifisch über Alzheimer-Demenz. Weitere spezifische Formen (vaskuläre Demenz, *n* = 4; Lewy-Körperchen-Demenz, *n* = 2; frontotemporale Demenz, *n* = 1) werden nur selten thematisiert.

### Zielgruppen und weiterführende Angebote (FF1)

Zunächst wird betrachtet, für welche intendierte Zielgruppe die Materialien aufbereitet sind. In 64 Materialien wurde mindestens eine Zielgruppe spezifisch adressiert. Dabei wurden Angehörige von Menschen mit Demenz (*n* = 43; 67,2 % der spezifizierten Materialien) am häufigsten angesprochen, gefolgt von Menschen mit Demenz selbst (*n* = 23; 35,9 %). Generell Interessierte (*n* = 22; 34,4 %) wurden häufiger als potenziell Betroffene (*n* = 17; 26,6 %) adressiert, Expert*innen und Ärzt*innen (*n* = 6; 9,4 %) und professionelles Pflegepersonal (*n* = 2; 3,1 %) wurden im recherchierten Material hingegen kaum angesprochen. Eine differenzierte Betrachtung der Angehörigen zeigt, dass Materialien am häufigsten für pflegende Angehörige (*n* = 33; 76,7 % der Angehörigenmaterialien) aufbereitet sind, nur selten für rein erlebende Angehörige, die nicht pflegen (*n* = 2; 4,7 %), in 8 Fällen (18,6 %) war nicht weiter spezifiziert, ob das Material für rein erlebende oder pflegende Angehörige gedacht ist.

Neben der Zielgruppe des Materials wurden auch Verlinkungen erfasst, die auf weiterführende Materialien verweisen. In 64 Materialien waren Verlinkungen enthalten, am häufigsten auch hier für Angehörige (*n* = 31; 48,4 % der verlinkenden Materialien) und für Menschen mit Demenz (*n* = 25; 39,1 %). Für Interessierte (*n* = 24; 37,5 %) wurde ebenfalls häufig weiteres Material empfohlen, für potenziell Betroffene (*n* = 16; 25,0 %) weniger und für professionelles Pflegepersonal und Expert*innen/Ärzt*innen (jeweils *n* = 4; 6,3 %) erneut am seltensten. Bei den Verlinkungen wurden für Angehörige konkretisiert ausschließlich weiterführende Informationen für aktiv Pflegende (*n* = 18; 58,1 % der Verlinkungen für Angehörige) angeboten, die übrigen Angehörigenverlinkungen wurden nicht konkretisiert (*n* = 13; 41,9 %). Zuletzt wurden die insgesamt 34 erwähnten weiterführenden Beratungs- und Informationsangebote erfasst (Tab. 2).

**Tab. 2:** Weiterführende Angebote in demenzbezogenen Informationsmaterialien

	*n*	%
*Gemeinnützige Organisationen/eingetragene Vereine/staatliche Institutionen*		
Persönliche telefonische Beratung	22	64,7
Persönliche Online-Beratung	5	14,7
Persönliche Vor-Ort-Beratung	2	5,9
Schulungen	1	2,9
*Privatwirtschaftliche Unternehmen*		
Schulungen	7	20,6
Persönliche telefonische Beratung	3	8,8
Persönliche Online-Beratung	2	5,9
Persönliche Vor-Ort-Beratung	0	0,0
*Ärztliche Beratungsleistungen*	3	8,8

*N* = 34. Strukturiert nach Anbieter*innen. Mehrfachnennung möglich

### Gesundheitsrelevante Faktoren (FF2a)

Insgesamt wurden Behandlungsoptionen (*n* = 89) in mehr Informationsmaterialien erwähnt als Risikofaktoren (*n* = 55; Tab. 3). Innerhalb der Materialien, die über Risikofaktoren informieren, wurden individuell beeinflussbare Risikofaktoren am häufigsten erwähnt (*n* = 53; 96,4 % der Beiträge mit Risikofaktoren), soziales Netzwerk und Gesellschaft spielen kaum eine Rolle. In Hinblick auf Behandlungsoptionen waren die Ebenen hingegen relativ ausgewogen verteilt, nahezu alle Materialien mit Behandlungsoptionen erwähnten Maßnahmen auf gesellschaftlicher Ebene (*n* = 82; 92,1 % der Beiträge mit Behandlungsoptionen).

**Tab. 3:** Anzahl und Anteil gesundheitsrelevanter Faktoren (Risikofaktoren und Behandlungsoptionen) in den Informationsmaterialien, unterteilt in 3 Ebenen

Ebene	Risikofaktoren		Behandlungsoptionen	
	*n*	%	*n*	%
Individuell	53	96,4	69	77,5
Soziales Netzwerk	8	14,5	66	74,2
Gesellschaft	1	1,8	82	92,1

Basis für %‑Angaben sind jeweils *n*_*Risikofaktoren*_ = 55, *n*_*Behandlungsoptionen*_ = 89. Mehrfachnennung möglich

Ferner wurde überprüft, ob bestimmte Risikofaktoren und Behandlungsoptionen auf den 3 Ebenen Individuum, soziales Netzwerk und Gesellschaft jeweils gemeinsam auftreten. Hier zeigte sich nur auf individueller Ebene ein signifikanter Effekt, hier treten Risikofaktoren und Behandlungsoptionen besonders häufig gemeinsam auf (χ^*2*^*(1)* = 5,53, *p* = 0,019, φ = 0,24). Auf den anderen beiden Ebenen treten sie eher unabhängig voneinander auf.

### Entscheidungsbezogene Faktoren (FF2b)

Formelle, juristische Maßnahmen (*n* = 36) werden in Informationsmaterialien häufiger thematisiert als informelle Maßnahmen (*n* = 29). Dabei behandeln die meisten Informationsmaterialien sowohl formelle als auch informelle Maßnahmen (*n* = 21), 8 Materialien fokussieren die informellen und 15 die formellen Maßnahmen; die Mehrheit (*n* = 56) weist nicht auf Maßnahmen hin (χ^2^(1) = 23,51; *p* < 0,001; φ = 0,49).

In den meisten Fällen wird unter formellen Maßnahmen auf *professionelle Beratungsangebote zu juristischen Maßnahmen* hingewiesen (*n* = 30; 83,3 % innerhalb der formellen Maßnahmen), gefolgt von *Vorsorgevollmacht* und Patient*innenverfügung (je *n* = 15; 41,7 %). Ferner werden die *frühzeitige Bewerbung um einen Platz in spezialisierten Einrichtungen* (*n* = 13; 36,1 %) und die *Betreuungsverfügung* (*n* = 11; 30,6 %) erwähnt. Weitere Maßnahmen, die induktiv erfasst wurden und nur jeweils einmal (2,8 %) erwähnt wurden, sind ein aufzustellender *Behandlungsplan,* der vorsorgliche Abschluss einer *Haftpflicht- und Hausratversicherung*.

Unter den informellen Maßnahmen wird in allen Fällen auf frühzeitige Gespräche mit Angehörigen (*n* = 29, 100 % innerhalb der informellen Maßnahmen) verwiesen, um bspw. Behandlungswünsche zu besprechen; seltener werden auch Gespräche mit Freund*innen (*n* = 9; 31,0 %) empfohlen.

Abschließend wurde überprüft, ob Materialien bestimmter Anbieter*innen tendenziell auf spezifische Maßnahmen hinweisen. Für die formellen Maßnahmen zeigten sich keine signifikanten Unterschiede (χ^2^(6) = 5,14; *p* = 0,526; φ = 0,23). Die informellen Maßnahmen dagegen unterschieden sich signifikant, Krankenkassen (*n* = 7) wiesen besonders häufig auf sie hin, ebenso staatliche Gesundheitsbehörden (*n* = 6) und Berufsverbände (*n* = 5). Pharmaunternehmen (*n* = 3) wiesen teilweise darauf hin. Bei den übrigen Anbieter*innen waren informelle Maßnahmen unterrepräsentiert (χ^2^(6) = 29,61; *p* < 0,001, φ = 0,55).

## Diskussion

Ziel der Studie war es, die Zielgruppen sowie kommunizierte gesundheitsrelevante und entscheidungsbezogene Faktoren in demenzspezifischen Informationsmaterialien inhaltsanalytisch zu untersuchen. Das dabei angewendete Verfahren der systematischen Stichprobenziehung kann bspw. für Angebotsevaluationen adaptiert werden, sie ist generisch auf verschiedene Gesundheitskontexte anwendbar.

Betrachtet man die in den untersuchten Materialien adressierten Zielgruppen, fällt auf, wie selten potenziell Betroffene angesprochen werden. Dies könnte daran liegen, dass diese Zielgruppe nicht sehr spezifisch definiert ist, da „potenziell Betroffene“ prinzipiell alle Menschen meint, die noch nicht eine Demenzdiagnose erhalten haben. Gleichzeitig enthalten auch Informationsangebote für die anderen Zielgruppen (z. B. Angehörige) risikobezogene und präventive Inhalte, also für potenziell Betroffene relevante Inhalte. Fraglich ist zudem, ob es überhaupt sinnvoll wäre, umfassend Materialien für potenziell Betroffene aufzubereiten, da dies gerade im Kontext Demenz bei einem Teil der Informationssuchenden auch dramatisierend wirken und Reaktanz auslösen könnte [28–31]. Für Menschen, die neu eine Demenzdiagnose erhalten haben bzw. noch in einem frühen Stadium sind, finden sich hingegen einige Materialien im Sample. Materialien für professionelles Pflegepersonal und Expert*innen sowie Ärzt*innen gibt es (im systematischen Sample) vergleichsweise selten. Dies scheint plausibel, da es für diese Zielgruppe Fachliteratur u. Ä. gibt und Expert*innen mutmaßlich auch spezifischer mit den entsprechenden Fachbegriffen suchen. Diese Studie hatte das Ziel, vorrangig Informationsmaterialien für Laien zu untersuchen bzw. das Rechercheverhalten von Laien zu simulieren, insofern war zu erwarten, dass die Zielgruppe der Expert*innen unterrepräsentiert ist.

Auffallend war, dass nur selten weiterführende Beratungs- und Informationsangebote erwähnt wurden (34 %). Interessanterweise werden hier die niederschwellig verfügbaren telefonischen Beratungsangebote am intensivsten beworben, die praktisch aber kaum genutzt werden [32]. Schulungen und persönliche (gemeinnützige) Vor-Ort-Beratungsangebote werden hingegen kaum beworben.

Behandlungsoptionen wurden häufiger erwähnt als Risikofaktoren. Interessanterweise zeigt sich ein ähnliches Verteilungsmuster auch in der Medienberichterstattung, die neben spezifischen Informationsmaterialien eine wichtige Quelle ist und daher vergleichend eingeordnet werden soll [33]. Dazu wird eine bestehende Studie zur demenzbezogenen Medienberichterstattung herangezogen [23]. Dort werden Risikofaktoren ebenfalls in rund 52 % aller demenzbezogenen Beiträge erwähnt, während Behandlungsoptionen in ca. 81 % genannt werden. Auch die Ebenen innerhalb der Risikofaktoren sind ähnlich verteilt, hier werden sowohl in der Berichterstattung als auch in den digitalen Informationsangeboten tendenziell individuelle Faktoren erwähnt. Unterschiedlich fallen hingegen die Behandlungsoptionen aus, in der Medienberichterstattung werden sie vor allem auf gesellschaftlicher Ebene verortet. In Informationsmaterialien werden Behandlungsoptionen auf gesellschaftlicher Ebene ebenfalls häufig genannt, aber auch individuelle und das soziale Netzwerk betreffende Optionen werden erwähnt.

Eine unausgewogene Darstellung von Risikofaktoren und zugehörigen Behandlungsmaßnahmen kann problematisch sein (siehe Einleitung). Einzelne Behandlungsmaßnahmen können nicht nachvollzogen werden, da die entsprechenden Risikofaktoren nicht bewusst sind und nicht umfassend erklärt wurden – und umgekehrt. Individuelle Risikofaktoren wurden meist mit entsprechenden Behandlungsoptionen genannt, also ausgewogen dargestellt. Dass Risikofaktoren auf gesellschaftlicher Ebene gar nicht thematisiert wurden, erscheint hingegen defizitär und problematisch. Eine sehr individualisierte Darstellung birgt die Gefahr einer Stigmatisierung, da fälschlicherweise vermittelt werden könnte, dass gerade Individuen für die Risikofaktoren verantwortlich sind und sie nicht auf gesellschaftlicher Ebene bestehen. Dies könnte auch eine (erlernte) Hilflosigkeit verstärken, die gerade bei Demenz relevant ist [34] – obwohl es evidenzbasierte präventive Empfehlungen gibt [4]. Dies stellt eine Herausforderung bei der adäquaten Informationsaufbereitung dar, da gerade die nicht modifizierbaren Risikofaktoren auf individueller Ebene (Alter, Geschlecht, Genetik) relevante Faktoren sind, trotzdem aber bis zu 40 % des Demenzrisikos modifizierbar sind und dies entsprechend betont werden sollte [4].

Tatsächlich sind diese Muster der Verantwortungsattributionen aus Informationsmaterialien und Medienberichterstattung ansatzweise auch in der Bevölkerung erkennbar, u. a. schätzen Menschen die Verantwortung für Behandlungsmaßnahmen insgesamt höher ein als die Risikoverantwortung [35].

Neben dargestellten Risikofaktoren und Behandlungsoptionen untersuchte die Studie auch erwähnte entscheidungsbezogene Faktoren, die präventiv ergriffen werden können, um demenzbezogene Entscheidungen zu unterstützen. Obwohl diese wichtig sind, um ohnehin belastende Entscheidungen abzufedern, werden sie in den Informationsangeboten nur selten adressiert. Gerade die Patient*innenverfügung und die Vorsorgevollmacht wären wichtige (formelle) präventive Maßnahmen, die auffallend selten erwähnt werden (in jeweils 15 Materialien). Die informellen Maßnahmen wurden sogar noch seltener erwähnt. Dabei ist bereits lange bekannt, dass klärende Gespräche mit Angehörigen und Freund*innen sowie entsprechende Dokumente (Advanced Care Planning) essenziell sind [36], um später mit (möglichst) gutem Gewissen einen mutmaßlichen Willen einschätzen zu können. Gerade gemeinnützige Vereine und Nichtregierungsorganisationen haben deutlich unterdurchschnittlich auf die entscheidungsbezogenen Maßnahmen hingewiesen, auch in Online-Gesundheitsportalen ist dieses Defizit erkennbar.

Für Praktiker*innen können folgende Implikationen aus der Untersuchung abgeleitet werden: In (digitalen) Informationsmaterialien könnten weiterführende Beratungsangebote stärker beworben werden. Zudem könnte eine kritische Reflexion angestoßen werden, warum die eigentlich sehr niederschwellig erreichbaren Info-Telefonnummern nicht genutzt werden, obwohl sie in den Materialien (vergleichsweise) häufig erwähnt werden.

Insgesamt könnten eigene Materialien inhaltlich darauf überprüft werden, ob Risikofaktoren und Behandlungsoptionen ausgeglichen präsentiert werden. So kann die Akzeptanz gegenüber den Inhalten weiter gefördert werden, außerdem werden eine mögliche Reaktanz oder erlernte Hilflosigkeit reduziert. Zudem könnten entscheidungsbezogene Maßnahmen als wichtige Präventionsmaßnahmen verstärkt in den Fokus gerückt werden.

### Limitationen und Ausblick

Eine wichtige Limitation betrifft die Suchstrategie. So war für einzelne Angebote nicht ersichtlich, wann sie erschienen oder überarbeitet wurden. Es könnte sich also um veraltete Informationen gehandelt haben, die in den Suchmaschinen trotzdem an hoher Stelle gelistet werden. Da über die systematische Stichprobenziehung aber dezidiert eine reale Suche simuliert werden sollte, wurden diese Angebote trotzdem aufgenommen, da sie in der Ergebnisliste auch real erscheinen würden. Ebenso könnten die verwendeten Suchstrings an sich limitierend sein. Obwohl sie sorgfältig ausgearbeitet und mit mehreren erfahrenen Forscher*innen und Anwender*innen kritisch reflektiert wurden, könnten andere Informationssuchende zu (leicht) anderen Rechercheergebnissen kommen, je nachdem wie sie online nach Informationen suchen. Diese Ergebnisse könnten in der Stichprobe verzerrt abgebildet oder unterrepräsentiert sein. Zukünftige Studien könnten spezifizierte Suchstrings anwenden, um das Angebot für bestimmte Zielgruppen oder Vorstadien einer Demenz konkreter zu erfassen.

Eine dritte Limitation betrifft die untersuchten Angebote. Offline-Angebote wurden nicht explizit berücksichtigt, nicht zuletzt, da es sehr aufwändig ist, diese repräsentativ zu beschaffen. Entsprechend gelten alle Implikationen aus dieser Studie nur für digitale Angebote, Offline-Angebote waren unterrepräsentiert. Zudem wurden audiovisuelle Angebote wie Videoplattformen, Podcasts oder eingebettete (Aufklärungs‑)Videos nicht untersucht, sondern ausschließlich textbasierte Angebote. Auch hier bietet sich ein Anknüpfungspunkt für zukünftige Forschung mit erweitertem Angebotspool.

Zuletzt ist anzumerken, dass die Daten bereits im Sommer 2021 erhoben wurden und die Inhalte und Angebote sich zwischenzeitlich verändert haben könnten. Eine stichprobenartige Prüfung weist auf keine relevanten Änderungen hin. Eine vollständige Neuerhebung für 2023 war aufgrund finanzieller und personeller Ressourcen jedoch nicht möglich. Hier könnte zukünftige Forschung anknüpfen, um Langzeitveränderungen zu identifizieren.

## Fazit

Im Rahmen der Studie konnte explorativ untersucht werden, wie demenzbezogene Informationsmaterialien aufbereitet sind. Die Anwendung der innovativen Methode hat sich als effektiv erwiesen, um das breite Spektrum an Materialien systematisch zu erfassen und eine repräsentative Stichprobe zu generieren.

Die Analyse legt nahe, dass die Darstellung von Risikofaktoren und Behandlungsmöglichkeiten ausgewogener gestaltet werden sollte, um ein umfassenderes Verständnis zu schaffen. Zudem könnte stärker auf gesellschaftliche Risikofaktoren eingegangen werden, um das Bewusstsein für deren Auswirkungen zu erhöhen. Auch die Informationen zu entscheidungsbezogenen Faktoren sollten umfassender vermittelt werden, um eine fundierte Entscheidungsfindung zu fördern.

## Footnotes

1 *Hinweis:* Das erst seit Januar 2023 bestehende Ehegattenvertretungsrecht als formelle Maßnahme wurde in dieser Studie nicht berücksichtigt, da es zum Zeitpunkt der Codierung noch nicht existierte.

2 Die recherchierten Materialien wurden Ende 2023 erneut stichprobenartig hinsichtlich inhaltlicher Aktualisierungen überprüft. Es konnten keine für die Analysen relevanten Änderungen identifiziert werden.
